# Identifying Potentially Avoidable Emergency Department Visits of Long-Term Care Hospital Residents in Korea: A Multicenter Retrospective Cohort Study

**DOI:** 10.1155/2019/7041607

**Published:** 2019-06-19

**Authors:** Keon Kim, Dong Hoon Lee, Ho Young Yune, Jung Hee Wee, Duk Ho Kim, Eui Chung Kim, Jee Yong Lim, Seung Pil Choi

**Affiliations:** ^1^Department of Emergency Medicine, Ewha Womans University Hospital, Seoul 07985, Republic of Korea; ^2^Department of Emergency Medicine, College of Medicine, Chung-Ang University, Seoul 06973, Republic of Korea; ^3^Department of Emergency Medicine, Hallym University Dongtan Sacred Heart Hospital, Hwaseong-si, Gyeonggi-do 18450, Republic of Korea; ^4^Department of Emergency Medicine, College of Medicine, Yeouido St. Mary's Hospital, The Catholic University of Korea, Seoul 07345, Republic of Korea; ^5^Department of Emergency Medicine, Eulji University, Seoul 01830, Republic of Korea; ^6^Department of Emergency Medicine, CHA University School of Medicine, Seongnam-si 13496, Republic of Korea; ^7^Department of Emergency Medicine, Seoul St. Mary's Hospital, Seoul 065691, Republic of Korea

## Abstract

The aims of this study were to investigate the reasons of transfers from long-term care hospitals (LTCHs) to emergency departments (EDs) of university hospitals in geriatric patients and to categorize the avoidable causes of these transfers. This retrospective multicenter study involved patients aged 65 years and older who were transferred from LTCHs to 5 EDs of university hospitals located in the metropolitan area of South Korea between January 2017 and December 2017. The expert panel reviewed and categorized the reason of transfers as avoidable or not. Moreover, we also investigated the number of patients with do-not-resuscitate (DNR) documents and the date these DNR documents were written. A total of 255,543 patients visited 5 EDs during the study period. Of these, 1,131 patients were from LTCHs. The number of potentially avoidable transfers was 168/1,131 (14.9%). The most common reason of avoidable transfers was noncritical diagnoses that could be assessed and managed in LTCHs (57.1%). There were 162 patients with DNR orders; of these, 12 had approved the DNR order before transfer. In conclusion, in Korea, potentially avoidable transfers could be reduced by managing noncritical diseases in LTCH and preparing advance care directives, including DNR orders, during admission to LTCH.

## 1. Introduction

In Korea, the number of the elderly population is rapidly increasing because of prolonged life expectancy and low birth rate. According to the Statistics Korea database, the rate of the elderly aged 65 years and older has increased from 10.2% in 2008 to 13.8% in 2017, and it is expected to become 37.8% by 2050 [1]. With increasing age, the medical demand also increases in this population. The rate of total medical expenses in the elderly aged 65 years and older is steadily increasing from 31.6% in 2010 to 36.8% in 2015. Additionally, the number of long-term care hospitals (LTCHs) has increased consistently from 714 in 2009 to 1,516 in 2017 [2]. An LTCH in Korea is a medical institution where doctors and nurses are stationed and where patients requiring long-term hospitalization and treatment due to senile diseases are mainly hospitalized. The elderly patients' total healthcare costs in LTCH are covered by the National Health Insurance (NHI) in Korea. According to the National Health Insurance Expenditure Trends Report by the National Health Insurance Review and Assessment Service [3], the total healthcare costs increased by 6.4% in 2015 compared to those in 2014, and one of the major causes was increased LTCH admission of the elderly patients. There are also nursing homes for elderly in Korea. A nursing home is a welfare facility that cares for rather than treats the elderly, whereas an LTCH is a “hospital” where patients can be treated or rehabilitated. Therefore, caregivers in nursing homes assist elderly patients in their daily activities, such as cooking, doing laundry, and physical activities such as excretion and bathing, especially those having difficulty in mobility due to senile diseases, such as dementia and stroke. Additionally, LTCHs have full-time working physicians, who are absent in nursing homes, and an outside physician visits twice a month [4].

LTCHs in Korea basically provide simple conservative treatments because of conflicting payment systems and lack of professional personnel and suitable facilities [5, 6]. Therefore, although LTCHs are medical institutions, practically only simple medical interventions, such as maintenance of existing medication and empirical medical treatment for newly occurring complications, can be performed in an LTCH. It is reasonable that patients in LTCHs are transferred to tertiary university hospitals when they have unstable vital signs or acquire new diseases or when their existing diseases worsen. These patients are assessed and treated in the emergency department (ED). However, some patients from LTCH are transferred unnecessarily to tertiary hospitals.

Unnecessary transfers can cause physical, mental, and economic distress to patients. Such transfers can also waste the limited medical resources of university hospitals and add to ED overcrowding in tertiary university hospitals. Therefore, there is a need to consider the potentially avoidable transfers from LTCHs. However, in Korea, there is a paucity of the data on patients who are transferred from an LTCH to the ED of tertiary university hospitals [7–11]. These studies showed the overall characteristics of geriatric patients transferred from LTCHs, but they did not consider the appropriateness of transfers. Thus, this study aimed to estimate the rate of potentially avoidable transfers from LTCHs to EDs of university hospitals in Korea and to categorize the causes of these transfers.

## 2. Methods

### 2.1. Study Setting and Participants

This multicenter, retrospective cohort study investigated all ED visits of LTCH patients aged 65 years and older between January 2017 and December 2017 in 5 tertiary university hospitals located in the metropolitan area of South Korea. The bed capacity of the enrolled hospitals was 620, 830, 850, 890, and 1,000 beds. Moreover, the number of patients visiting the ED of these hospitals annually ranges from 40,000 to 70,000.

### 2.2. Data Sources

#### 2.2.1. National Emergency Department Information System

Data of patients who visited EDs between January 2017 and December 2017 were extracted from the National Emergency Department Information System (NEDIS) of Korea, which is a nationwide government system that has been in operation since 2003 and collects data from more than 150 Korean emergency centers.

We analyzed the following variables provided by the NEDIS: patient demographic information (age, sex), triage acuity (Korean Triage and Acuity Scale [KTAS]), main reasons for ED visit (medical or nonmedical cause), primary diagnosis in the ED, and disposition at the ED (returned to an LTCH, admitted to transferred hospital, admitted to another hospital, left before treatment completed, deceased). The KTAS is a Korean triage system that categorizes patients by severity from 1 to 5, with 1 being the highest.

#### 2.2.2. Electronic Medical Record Review

After extracting the patients' data in the NEDIS database, we reviewed the patients' electronic medical review (EMR) to determine the patients' detailed diagnosis in the ED and the reason for transfers. We also assessed other variables, such as the following: causes of LTCH admission, whether the patients were transferred from an LTCH, whether they signed the do-not-resuscitate (DNR) order, and the point in time when the decision of DNR was taken.

### 2.3. Outcome Measurements

The primary outcome was the number of avoidable transfers. To develop the available tools that identify avoidable transfers, we (1) developed a conceptual framework, (2) developed the criteria of potentially avoidable transfers, (3) recruited and trained expert panels who then performed chart reviews, and (4) analyzed the appropriateness of transfers.

The secondary outcome was the number of patients who have already signed or provided informed consent for the DNR order while still in the LTCHs, and the number of patients who signed the DNR form at the ED or after ED admission. Patients who have already signed the DNR order can experience natural death in an LTCH if their condition deteriorates while undergoing conservative treatments. These data were obtained by the panel through a chart review.

#### 2.3.1. Conceptual Framework and Development of the Criteria of Potentially Avoidable Transfers

We developed our conceptual framework and the criteria of potentially avoidable transfers based on the medical literature. We reviewed several previous studies [12–19] and analyzed the appropriateness of transfers. Additionally, from a number of available tools that identify avoidable transfer criteria, we selected the tool developed by Morphet et al. [19], who validated this tool that is relevant to the Australian health context. We modified and developed the criteria considering the current status of LTCHs in Korea, specifically when it comes to human and equipment resources, procedure availability, quality of evaluation, and acute illness treatment. As a result, the criteria for potentially avoidable transfers were divided into the following five categories: (1) presence of noncritical diagnoses that could be assessed and treated in an LTCH, such as cellulitis or urinary tract infection (UTI), provided that patients have no signs of systemic toxicity and have no uncontrolled comorbidities and the condition can usually be managed with oral antimicrobials; (2) family member/members who refused further evaluation and treatment in the ED; (3) already-known advance care directives (including DNR); (4) simple procedure such as replacement of urinary catheter because of failure of insertion in an LTCH; and (5) minor traumas not requiring ED assessment, such as abrasions, bruises, skin lesions not requiring sutures, and minor burns covering only a small area of skin.

#### 2.3.2. Expert Panel Reviewer Recruitment

We recruited an expert panel composed of three board-certified emergency medicine physicians, who have more than 10 years of experience, to review the patients' EMR. The panel reviewed previous studies and made a consensus about the criteria for potentially avoidable transfers. On the basis of the results on the patients' EMR, the panel decided on whether the transfer from an LTCH to the ED could be avoidable or not.

### 2.4. Statistical Analysis

All statistical analyses were performed using IBM SPSS version 23.0 (IBM Corp., Armonk, NY, USA). We entered descriptive statistics for the numbers and percentages of the study population. The chi-squared test was used to compare the categorical variables between groups. Statistical significance was set at* p-*value less than 0.05.

### 2.5. Ethics Statement

This study was approved by the institutional review boards of each hospital, and the need for patient informed consent was waived.

## 3. Results

**Figure 1:**
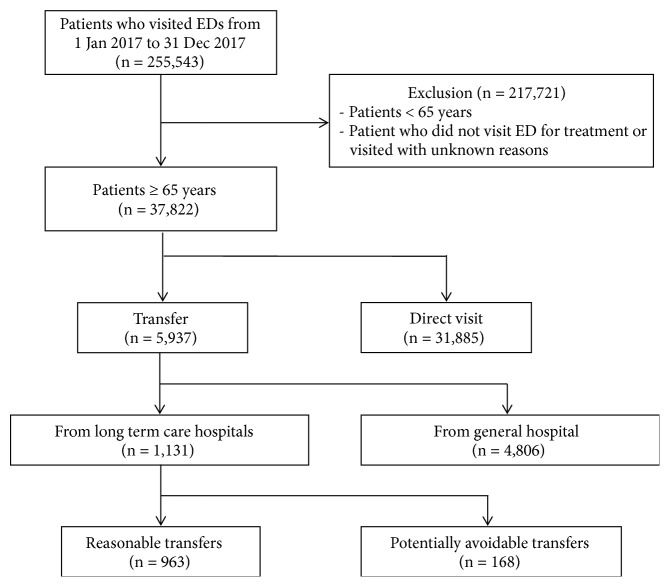
The flowchart of the study population. ED: emergency department.

A total of 255,543 patients presented to the 5 EDs in 2017. Of these, 37,822 (14.8%) patients were aged 65 years and older. The number of patients transferred from LTCHs to EDs was 1,131, which represents 3.0% of all ED patients aged 65 years and older. Of these, 168 (14.9%) patients were categorized as the potentially avoidable transfer group, whereas 963 (85.1%) patients were categorized as the reasonable transfer group (Figure 1).

### 3.1. General Characteristics of LTCH Patients Transferred to the ED of University Hospitals

The baseline characteristics of the study population are shown in Table 1. Age, sex, triage acuity, main reasons for ED visits (medical or nonmedical), primary diagnosis in the ED (trauma or nontrauma), and frequency of admission to the general ward or intensive care unit were not statistically different between the reasonable and potentially avoidable transfer groups (Table 1). The reasons of LTCH patient admissions were different between the potentially avoidable and reasonable transfer groups (p=0.043). Cerebrovascular disorder and dementia were the most common causes in both groups, which were followed by orthopedic injuries, malignancies, and end-stage kidney disease in the reasonable transfer group and malignancies, orthopedic injuries, and generalized weakness in the potentially avoidable transfer group. After transferring to the ED, the primary diagnoses (nontrauma) were different between the two groups. The potentially avoidable transfer group was more frequently coded as no disease found (15.3% vs. 1.4%, p<0.001).

Regarding their disposition in the ED, 98 (58.3%) and 189 (19.6%) patients of the potentially avoidable and reasonable transfer groups, respectively, returned to their LTCH without hospital admission (p<0.001). In contrast, the rate of patients who were admitted to university hospitals was much higher in the reasonable transfer group (75.3% vs. 4.8%, p<0.001) than in the potentially avoidable group. There was no significant difference in the percentage of admission to the general ward or intensive care unit between the 2 groups (p = 0.096).

### 3.2. Potentially Avoidable Reasons for ED Transfer

Of the 168 potentially avoidable transfers, the most common reasons were the following: noncritical diagnoses that could be assessed and treated in an LTCH (96/168, 57.1%), family member/members who refused further evaluation and treatment in the ED (58/168, 34.5%), already-known advance care directives (including DNR) (6/168, 3.6%), simple procedures that could be performed in an LTCH (5/168, 3.0%), and minor traumas not requiring ED assessment (3/168, 1.8%, Table 2). Regarding the noncritical diagnosis, there were cases of urinary tract infection (28 patients), nonspecified diagnosis (23 patients), upper respiratory infection (22 patients), gastrointestinal problems including gastritis or enterocolitis (8 patients), cellulitis (5 patients), herpes zoster (3 patients), arthritis (2 patients), epistaxis (2 patients), conjunctivitis (2 patients), and hypoglycemia (1 patient).

### 3.3. Number of Patients with Do-Not-Resuscitate (DNR) Order according to the Location Where Informed Consent for DNR Was Obtained

DNR orders were found in 162 (14.3%) patients aged 65 years and older who were transferred from an LTCH to the ED (Table 3). In the reasonable transfer group, 6 (3.7%), 20 (12.3%), and 87 (53.7%) patients signed the DNR order in the LTCH, ED, and ward or intensive care unit, respectively, whereas in the potentially avoidable transfer group, 6 (3.7%), 36 (22.2%), and 7 (4.3%) patients signed the DNR order in the LTCH, ED, and ward or intensive care unit, respectively.

## 4. Discussion

In this study, the rate (14.9%) of transfers from an LTCH to the ED of a university hospital in patients aged 65 years and older was potentially avoidable. However, this rate is lower than that in the previous studies [12, 17, 19, 20], which reported that 31%–53% of all ED transfers from care homes are avoidable. Codde et al. [12] and Morphet et al. [19] included the transfer for simple suturing of soft tissue injury during office hours and insertion of failed urinary catheter despite repeated attempts or replacement of gastrostomy as potentially avoidable transfers, which was contrary to the results in our study. In this study, we did not categorize these reasons as potentially avoidable transfers in consideration of the LTCH situation in Korea. Therefore, the frequency of avoidable transfers in our study was significantly low compared to previous studies.

In Korea, LTCHs have difficulty in performing such procedures, including simple sutures, because of the current system in these institutions. The most frequently mentioned difficulty by LTCH personnel was that they could barely provide high-quality medical services since the fixed-sum medical fee per day payment system was implemented in 2008. A previous nationwide survey including 104 chief executive officers from LTCHs revealed problems of this fee system [21]. Another Korean study also reported that some LTCHs regularly sent patients to other hospitals and took a medicine from that hospital to reduce the medical expenses, even when patients paid their medical fee in LTCH [22]. This payment system applies the fixed cost of hospitalization per day depending on the disease and functional condition of inpatients. In this system, LTCHs are forced to minimize the number of their healthcare personnel and downgrade the management efforts and medications.

Several studies investigated the service quality and problem of LTCHs. The level of medical service quality was higher in LTCHs with a lower patient: nurse ratio and a lower turnover rate of nursing personnel [23]. Low turnover was an important factor in providing quality healthcare management, which was likely due to the provision of more consistent treatments and effective communications [24, 25]. However, the mandatory number of healthcare personnel required in LTCH is lower than that in the general hospitals in Korea. For a general hospital, 1 doctor per 20 hospitalized patients and 1 nursing staff (nurse, nurse assistant) per 2.5 patients are needed, whereas for LTCHs, 1 doctor per 40 patients and 1 nursing staff per 6 patients are required. Thus, increased manpower within LTCHs could be one of the solutions to prevent potentially avoidable transfers of LTCH patients.

In this study, the most common reason for the potentially avoidable transfers was noncritical diagnoses that could be assessed and treated in an LTCH, which accounted for 96/168 (57.1%) of avoidable transfers and 96/1131 (8.5%) of the total transfers from LTCHs in patients aged 65 years and older (Table 2). These noncritical diagnoses included the following: simple urinary tract infections without fever and with stable vital signs requiring only oral antibiotic therapy, cellulitis requiring only oral antibiotics and conservative treatment with mild localized symptoms, and epistaxis requiring only an application of nasal packing. These conditions could be empirically managed by simple medication treatment without specific evaluation. Therefore, if physicians in an LTCH could manage these noncritical diseases, the number of potentially avoidable transfers might be reduced.

DNR orders were found in 162 of the 1131 patients transferred from LTCHs to the EDs, but only 12 patients signed the DNR order at the LTCH before transfer (Table 3). More patients provided informed consent for DNR order at the ED than at the LTCH. In Korea, the law ‘act on hospice and palliative care and decisions on life-sustaining treatment for patients at the end' was implemented in August 2017 [26]. Before this law was implemented, patients usually make advance care directives including DNR in the ED after the transfer, which is consistent with the result of this study. Therefore, a clear communication about care plans is needed between the physicians and patients in LTCHs at the time of LTCH admission. Another reason of avoidable transfer was represented by the refusal to treatment by the family at ED (Table 2). This problem also could be resolved by the clear communication about advance care directives between LTCH physicians and patients or their families.

Preventing potentially avoidable transfer is related to reduced overcrowding of the ED and its adverse effects. Given that elderly patients tend to have cognitive impairment, they are at risk of falling, depression, functional decline, and sensory disturbance, and they are also prone to being prescribed with multiple medications [27, 28]. These characteristics complicate the evaluation and treatment of the elderly in the ED, requiring complex and lengthy clinical evaluations. Therefore, the potentially avoidable transfers of the elderly patients may be one of the causes of ED overcrowding. Furthermore, some studies have identified the effect of high occupancy and access block in the ED as causes of adverse patient outcomes, high mortality rates (20%–30%), increased risk of errors, prolonged inpatient length of stay, delayed critical care time, and hospital readmission [29, 30].

Our study has some limitations. First, a major limitation of this study was its retrospective design. Given that researchers retrospectively reviewed the EMR, there was an inevitable risk of bias. Second, although this was a multicenter study, only 5 EDs of the university hospitals around the metropolitan area of Korea and 75 LTCHs were analyzed. Therefore, this could not reflect the characteristics of the rural area and could have affected the generalizability of the results. Third, the opinion of an “expert panel” was subjective, and the expert panel consisted of only emergency physicians. Finally, this study included patients who were transferred from LTCHs to EDs, but not patients from nursing homes. For these reasons, our results should be interpreted cautiously.

## 5. Conclusions

This study showed that the rate of potentially avoidable transfers from LTCHs to EDs of university hospitals was 14.9% in Korea. These avoidable transfers could be reduced by managing noncritical diseases and preparing advance care directives, including DNR order, during LTCH admission. Additionally, increasing the healthcare provider-patient ratio at LTCHs and off-loading some provider responsibility could be solutions at the policy and infrastructure level.

## Figures and Tables

**Table 1:** General characteristics of the study population transferred from LTCHs to EDs.

Characteristics	Reasonable	Potentially avoidable	*p*-value
	(N=963)	(N=168)	
Age, years, n (%)			0.131
65–74	213 (22.1)	26 (15.5)	
75–84	461 (47.9)	84 (50.0)	
≥85	289 (30.0)	58 (34.5)	
Sex, n (%)			0.234
Male	449 (46.6)	70 (41.7)	
Female	514 (53.4)	98 (58.3)	
Triage category (KTAS), n (%)			0.372
1	34 (3.5)	8 (4.8)	
2	220 (22.8)	36 (21.4)	
3	557 (57.8)	88 (52.4)	
4	120 (12.5)	29 (17.3)	
5	32 (3.3)	7 (4.2)	
Common causes of LTCH admissions, n (%)			0.043
Cerebrovascular disorders	400 (41.5)	62 (36.9)	
Dementia	141 (12.7)	28 (18.4)	
Orthopedic injuries	106 (11.0)	15 (8.9)	
Malignancies	71 (7.4)	20 (11.9)	
End-stage kidney disease	69 (7.2)	5 (3.0)	
Generalized weakness	68 (7.1)	11 (6.5)	
Other trauma	30 (3.1)	8 (4.8)	
Congestive heart failure	20 (2.1)	4 (2.4)	
Chronic obstructive pulmonary disease	14 (1.5)	2 (1.2)	
Vegetative state	15 (1.6)	0 (0)	
Others	29 (3.0)	11 (6.5)	
Main reasons for ED visit, n (%)			0.317
Medical cause	884 (91.8)	158 (94.0)	
Nonmedical cause	79 (8.2)	10 (6.0)	
Primary diagnosis in the ED, n (%)			0.097
Trauma	60 (6.2)	5 (3.0)	
Non-trauma	903 (93.8)	163 (97.0)	
Specific diagnoses (non-trauma)			<0.001
Respiratory tract	311 (34.4)	38 (23.3)	
Urologic	115 (12.7)	27 (16.6)	
Gastrointestinal	165 (18.3)	17 (10.4)	
For procedure	73 (8.1)	10 (6.1)	
Cardiovascular	52 (5.8)	7 (4.3)	
Cerebrovascular	56 (6.2)	6 (3.7)	
Fluid and electrolyte disorder	20 (2.2)	3 (1.8)	
Sepsis	47 (5.2)	3 (1.8)	
Cardiopulmonary arrest	2 (0.2)	3 (1.8)	
No disease found	13 (1.4)	25 (15.3)	
Others	49 (5.4)	24 (14.7)	
Disposition in the ED, n (%)			<0.001
Returned to an LTCH	189 (19.6)	98 (58.3)	
Admitted to this hospital	730 (75.8)	37 (4.8)	
GW/ICU: n (%)			0.096
General ward	475 (65.1)	29 (78.4)	
Intensive care unit	255 (34.9)	8 (21.6)	
Admitted to another hospital	22 (2.3)	17 (10.1)	
Left before treatment completed	18 (1.9)	9 (5.4)	
Deceased	4 (0.4)	7 (4.2)	

*LTCH*: long-term care hospital, *ED*: emergency department, *KTAS*: Korean Triage and Acuity Scale, *GW*: general ward, *ICU*: intensive care unit.

**Table 2:** Potentially avoidable reasons for ED transfer.

	n	%
Potentially avoidable ED transfers (N=168)		
Non-critical diagnosis – assessment in an LTCH would be appropriate	96	57.1
Family member/members who refused further evaluation and treatment in the ED	58	34.5
Already-known advance care directives (including DNR)	6	3.6
Simple procedure	5	3.0
Minor trauma – ED assessment not required	3	1.8
Reasonable ED transfers (N=963)		
Signs of being systemically unwell – suitable observations cannot be provided	568	59.0
No response to treatment in an LTCH	103	10.7
Procedure unable to be performed in an LTCH	83	8.6
Abnormal results of laboratory or radiological examinations performed in an LTCH	83	8.6
Suspicion of cerebral event with neurological changes	73	7.6
History of trauma with suspected fracture	37	3.8
Family request for ED transfer	12	1.2
Open wound with suturing required	4	0.4

*ED*: emergency department, *LTCH*: long-term care hospital, *DNR*: do-not-resuscitate.

**Table 3:** Locations where informed consent for DNR was obtained.

Location	Reasonable	Potentially avoidable
	(n=113)	(n=49)
LTCH	6 (3.7)	6 (3.7)
ED	20 (12.3)	36 (22.2)
Ward or ICU	87 (53.7)	7 (4.3)

*DNR*: do-not-resuscitate, *LTCH*: long-term care hospital, *ED*: emergency department, *ICU*: intensive care unit.

## Data Availability

The data used to support the findings of this study are available from the corresponding author upon request.
